# Influence of hormones and HIV infection on viral transport

**DOI:** 10.1186/1742-4690-9-S2-P207

**Published:** 2012-09-13

**Authors:** DJ Stieh, SA Allan, SA Shukair, GC Cianci, AJ Fought, A French, TJ Hope

**Affiliations:** 1 Northwestern University, Chicago, IL, USA; 2 Ruth M. Rothstein CORE Center, Chicago, IL, USA

## Background

Inhibiting transport of virions within the female reproductive tract is an attractive mechanism for transmission prevention. The mucosal environment varies with menstrual cycle and concurrent bacterial vaginosis (BV). Previous studies of transport within mucosal samples have focused on cervico-vaginal samples in exclusion. Severe BV corresponds to increased incidence of female-to-male HIV-1 transmission although the mechanism remains unclear.

## Methods

We have established a cohort of HIV +ve and –ve women to be longitudinally studied for correlates of inhibited transport phenotypes. Cervical and cervico-vaginal mucus samples (CM and CVM, respectively) are collected, along with mucosal antibodies, vaginal smears, hormone levels, viral load, T cell monitoring panels as well as medical and behavioral history. Viral transport assays employ a diverse panel of viral isolates, testing clade specific effects. As of this interim analysis, over 60 study subjects have been recruited and repeat sampling is beginning.

## Results

The hormone profile demarcating menstrual cycle phase correlates strongly with particle movement. In CVM, the nature of viral interactions with their environment changes; lowest progesterone to estradiol ratio corresponds with hindered diffusion. Non-reactive similarly sized PEGylated beads have freely diffusive behavior throughout the menstrual cycle. Cycle classification into follicular, mid-cycle and luteal periods demonstrates that CVM in the luteal phase is, unexpectedly, most permissive to viral transport. Severe BV has modest effects on pH of CVM and no effect on CM. This is reflected in viral transport characteristics whereby the magnitude and nature of movement is invariant during severe BV relative to healthy flora.

## Conclusion

It is unlikely that the mechanisms of increased transmission with BV are related to virus diffusing more freely within mucus. Correlates of hindered diffusion are not restricted to adaptive immune responses. This study begins to reveal the significance of immune correlates and hormone profiles on HIV-1 transmission mechanisms and transport in the female reproductive tract.

